# Management of neonatal retro-auricular embryonal rhabdomyosarcoma – Case report

**DOI:** 10.1016/j.ijscr.2020.09.076

**Published:** 2020-09-12

**Authors:** M. Jean-Christophe Roubaud, Julien Prevot, Jean-Christophe Leclere, Charlotte Mistretta, Emmanuel Mornet, Rémi Marianowski

**Affiliations:** CHRU de Brest, Department of Otolaryngology, Head & Neck Surgery, Brest, France

**Keywords:** Rhabdomyosarcoma, Retro auricular, Children, Malignant tumor

## Abstract

**Introduction:**

Rhabdomyosarcoma (RMS) arises from mesenchymal cells committed to skeletal muscle. It is the most frequent soft-tissue sarcoma in children and makes up 5% of all pediatric malignant tumors. In this population, there are two main histological forms, which are the embryonal or the alveolar RMS. The retro auricular location is extremely rare. We present 2 cases of children with left retro-auricular RMS occurring at a very early stage of post natal development.

**Case reports:**

Two children were included in the RMS 2005 Protocol. The first child, aged 5-days, was managed by surgical resection in two steps after a previous incomplete resection which was followed by 8 chemotherapy cycle. The second, aged 3-days, was managed by surgical resection of the tumor bed, completed by 8 chemotherapy cycle. On regular follow up for over 6 years after the diagnosis, both patients are free of recurrence.

**Discussion:**

RMS is a fast-growing malignant and aggressive tumor originating from skeletal muscle. It occurs in the first decade of life and is associated with genetic conditions. With an unusual clinical presentation and anatomical location, both benign and malignant tumors can be suspected. Diagnosis can be performed with CT-scan or MRI and confirmed by biopsy. The treatment is based on chemotherapy followed by radiotherapy or surgical resection. In our two patients, the treatment program achieved complete disease control, with good prognosis especially because of anatomical location as well as early diagnosis.

## Highlights

• RMS is a fast-growing malignant and aggressive tumor originating from skeletal muscle.
• Diagnosis can be performed with CT-scan or MRI and confirmed by biopsy.
• The treatment is based on chemotherapy followed by radiotherapy or surgical resection.

## 1 Introduction

Rhabdomyosarcoma (RMS) is a malignant tumor of striated muscle. It is the most frequent soft-tissue sarcoma in children and represents 5% of all pediatric malignant tumors [1]. RMS arises from immature mesenchymal cells that are committed to skeletal muscle differentiation [2]. In the pediatric population, there are two main histological forms which are the embryonal and the alveolar RMS.

While the anatomical location of the primary tumor is variable, approximately 30% of all pediatric RMSs occur in the head and neck. Outer ear locations are considered extremely rare, thus making the early diagnosis challenging. Sometimes, inflammatory and infectious presentation can delay diagnosis and lead to unsuitable primary management [1,3].

Treatment of non-metastatic RMS follows the EpSSG RMS 2005 protocol [4], with a subgroup classification based on multiple risk factors. This tumor has been regularly described as having a poor prognosis, with patients succumbing to incurable local disease rather than distant metastasis [5]. We report 2 new cases of congenital retro auricular location and discuss treatment modalities according to existing international guidelines.

## 2 Presentation of case

A retrospective analysis was performed on files of 2 patients followed for retro auricular RMS in our department over a 6-year period from August 2011 to June 2019. Consent was obtained for both patients. The purpose of this retrospective analysis was to describe the diagnosis, management and survival rate of both children with retro auricular embryonal RMS. It emphasizes both clinical and radiological findings of this rare condition.

The extent of disease was determined by clinical examination, supplemented by radiological imaging including CT, PET-CT and MRI.

The tumor, nodes and metastasis classification of pediatric soft-tissue sarcomas (IRS) of the Union for International Cancer Control (UICC) allowed the cancer staging. Survival rate was determined from the end of treatment and for both children.

### 2.1 Patient #1

A 3-month-old girl presented with a left retro auricular mass that increased in size in a period of 3 months. This mass was discovered at post natal day 5 and was watchfully followed by the GP. There was no relevant family history. Examination revealed a slowly enlarging left retro auricular swelling without inflammatory or general sign. There was no history of trauma nor infection.

**Fig. 1:**
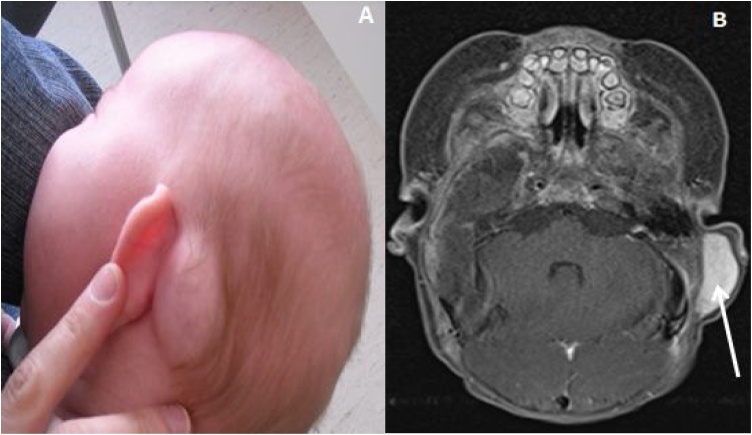
Clinical photograph of Patient #1, with the left retro auricular primary lesion (A) confirmed with axial post contrast MRI of head, fat-saturated, T1-weighted showing moderately intense (white arrow) and heterogeneous enhancement of the mass (B).

A temporal MRI was performed and discovered an oval mass (3 cm long axis) showing moderately intense and heterogeneous enhancement of the mass in post contrast T1 fat-saturated sequence (Fig. 1). A lymphangioma was suspected.

**Fig. 2:**
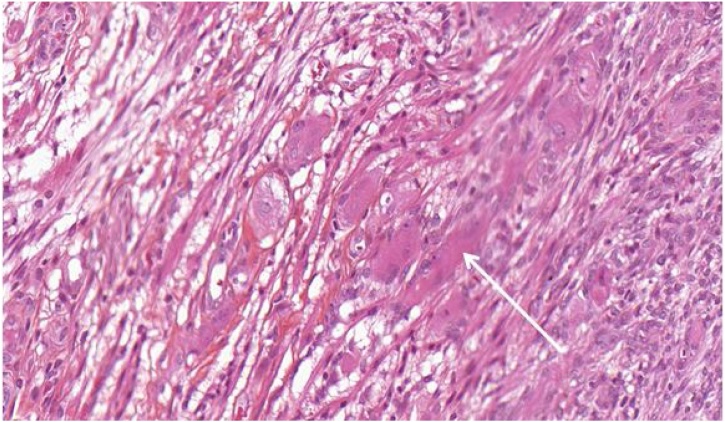
Patient #1, Frequent mitoses. Intense cytoplasm with voluminous streaks of muscle differentiation. Abnormally high proliferative activity (white arrow). Uneven size of cell nuclei (40x, HE stain).

A biopsy performed under general anesthesia was realized without anatomopatholigal result. Surgical resection was then performed. Macroscopic examination found a 35 × 27 × 16 mm well-delineated non-cystic lesion. Fetal rhabdomyoma was first diagnosed. On immunohistochemistry, about 30% of cells were myogenin positive but more heterogeneous cells were desmin positive. In contrast to rhabdomyome, the degree of orderly zonation was not achieved. There was mild but undoubted nuclear atypia and quite frequent mitotic figures. In this case, an embryonal RMS was finally diagnosed 3 months after surgical resection (Fig. 2).

Brain and temporal MRI, head and neck and thoracic contrast-enhanced CT-scan, PET-CT and myelogram were performed. Brain MRI showed a 6 mm left retro-auricular nodular lesion but cerebral parenchyma was normal. There wasn’t any cervical lymph node or metastatic extension on chest CT-scan or PET-CT. A complete resection was performed. The lesion was classified IRS I according to the classification of UICC.

The multidisciplinary team recommended implementing the EpSSG RMS 2005 lower risk group, subgroup A protocol [4]. The patient received 8 cycles of VA (Vincristine, Actinomycin) for 4 months. Radiation therapy was not considered as the mass was completely removed and histopathology was not alveolar type.

Regular follow up was established with clinical examination every 6 weeks and temporal and brain MRI every 3 months during the first year.

Then, during the 2nd and the 3rd years, the same follow up was performed every 4 months. During the 4th and the 5th years an annual clinical examination with brain and temporal MRI was performed. Beyond the 5th year, clinical examination was performed annually without MRI. After regular follow up for over 6 years after the diagnosis, the patient is free of recurrence.

### 2.2 Patient #2

An 18-month-old girl presented with a left retro auricular cyst, noticed during her first days of life and followed by the GP.

**Fig. 3:**
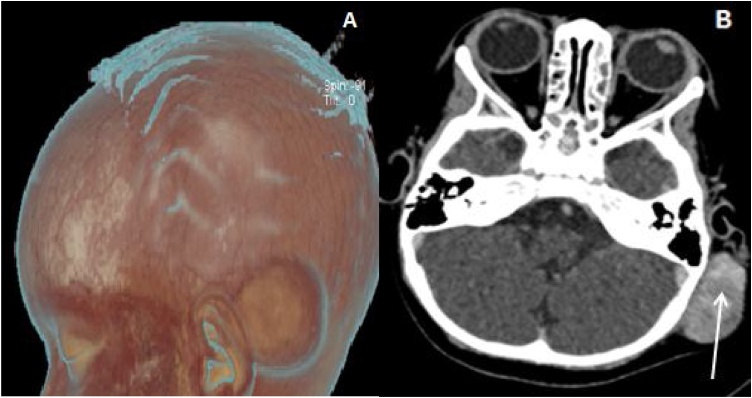
Patient #2, Radial reconstruction on CT-scan (A) and non-contrast CT-scan of head (B) showed a left retro auricular heterogeneous lesion (white arrow) measured at 41 mm as larger diameter, richly vascularized without bony involvement.

An ultrasound was initially performed and found a non-throbbing and non-adherent vascularized hypo echoic mass compatible with hemangioma. A CT-scan was realized to eliminate bone damage or vascular malformation. CT-scan showed a 41 × 36 mm left highly vascularized retro auricular tumor regardless of malignancy (Fig. 3).

**Fig. 4:**
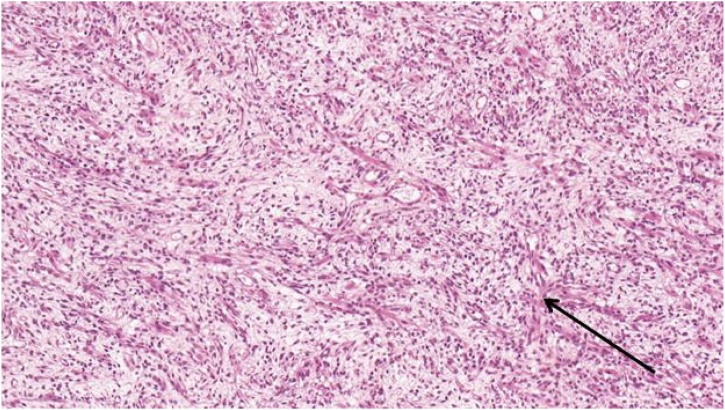
Patient #2, Dense cell proliferation with varying size of cells. Variable cytonuclear atypies. Frequent mitoses (black arrow). In some cytoplasms, eosinophil coloration with sometimes a draft of streaks (20x, HE stain).

Surgical resection was performed by retro auricular approach. Under general anesthesia, with patient in supine position, head turned to the right, a rhombic retro auricular incision was performed. Wide local excision of this vascularized tumor developed above the periosteal plan was successfully performed. Histopathological examination revealed embryonal RMS (Fig. 4).

Brain and temporal CT-scan found a subcutaneous left retro auricular enhanced-contrast tumor extended to occipital region.

A PET-CT was performed and found a bilateral latero-cervical hyper metabolism in IIA area according to the Robbins’ classification [11]. MRI found a left subcutaneous contrast enhancement in retro auricular, parapharyngeal, carotid, parotid and latero-cervical areas with a weak signal on T2 and FLAIR sequences. A right sub-maxillary lymph node (area IIA) 8 mm in small diameter in the axial plane and 12 mm large diameter in the coronal plane was assessed.

Revision surgery was performed with left cervical node dissection. No metastatic lymph node was diagnosed.

The tumor was classified IRS I according to the classification of UICC.

The multidisciplinary team recommended implementing the EpSSG RMS 2005 lower risk group, subgroup A protocol [4]. The patient received 8 cycles of VA without radiation.

A regular follow up was established according to the protocol of patient #1. We noticed a good tolerance to chemotherapy. After 60 months of follow up the patient is free of recurrence.

## 3 Discussion

RMS is a fast-growing malignant and aggressive tumor originating from skeletal muscle. RMS occurs in the first decades of life and is associated with genetic conditions like neurofibromatosis or Li Fraumeni syndrome [8]. It is the most common soft tissue sarcoma in children representing 5% of all pediatric malignant tumors. The main histologic subtypes are embryonal (60%) or alveolar (20%) [1]. Embryonal subtype is considered at low or standard risk, whereas the alveolar subtype is associated with poorer prognosis [2]. Retro auricular embryonal RMS has never been described to our knowledge.

Presenting symptoms varied patients and to the site of the disease. Orofacial RMS can be characterized by a painless cutaneous node or retro auricular swelling without inflammatory sign, as our cases. Because of non-specific presenting symptoms, they are frequently misdiagnosed and treatment can be delayed by several months.

With an atypical aspect, clinical presentation and unusual location, benign tumors, such as glomus tumor, hemangioma, pilomatrixoma, or malignant tumors, such as liposarcoma, chondrosarcoma, neuroepithelioma, extra skeletal Ewing’s sarcoma, juvenile fibromatosis or malignant lymphoma can be suspected [6,7]. RMS is a congenital tumor. Prenatal diagnosis by magnetic resonance imaging or ultrasound can be performed [9]. Skelton and Goodwin reported a neonate with intra oral embryonal RMS diagnosed on antenatal ultrasound scan. Prenatal MRI can be used to provide, accurate diagnosis of fetal masse.

Diagnosis can be suspected with CT-scan or MRI and confirmed by biopsy [8]. CT-scan assesses temporal bone cortex destruction [1]. MRI provides a better assessment of extension, lesion size or lymph node involvement. Imaging has a key role in the initial staging, the preoperative planning and allows early detection of recurrence during the follow up [6].

On CT-scan, RMS is a bulky tumor with multiple degrees of heterogeneous attenuation. On MRI, RMS is seen like a nonspecific low or isointense signal on T1-weighted sequences and high signal on T2-weighted images. In our case, differential diagnosis based on imaging were suspected because of characteristics comparable to a variety of other lesions (lymphangioma and hemangioma). Extension assessment can be completed by PET-CT, bone scintigraphy, chest CT-scan and osteomedullary biopsy to screen for second locations and evaluate metastatic extension. Metastasis of RMS is by hematogeneous, direct and lymphatic routes [8].

Histopathological examination only permits definitive diagnosis. RMS can be classified as embryonal, alveolar or pleomorphic type. The embryonal type is the commonest lesion (60%) whereas the alveolar type has the worst prognosis. Embryonal RMS is characterized by small round blue cells, like neuroblastoma or Ewing’s sarcoma, with primitive spindle cells and a myxoid background.

In case #1, 30% of cells were myogenin and heterogeneous desmin positive with nuclear atypia and frequent mitotic figures. Thus an embryonal RMS was diagnosed.

Immunocytochemistry, including staining for desmin, smooth muscle actin and myogenin confirmed the diagnosis [3]. There are translocations which result in alteration of biological activity at the protein level and influence behavior of tumor by impacting the control of cell growth, apoptosis, differentiation and motility. The translocations involve two PAX genes (PAX3 and PAX7 on chromosome 2 and 1) [8].

The intensity and duration of treatment for RMS is tailored on prognosis factors used to define risk groups. In Europe, a stratification system takes into account histologic type (alveolar or not), initial post- surgical status (complete, macro- or microscopically incomplete resection), location (favorable or not), lymph node invasion, patient age (less or greater than 10 years) and tumor size (less or greater than 5 cm). The European paediatric Soft Tissue Sarcoma Study Group (EpSSG) defined four groups: low risk, standard risk, high risk and very high risk [4]. This classification was used to define the treatment to our patients. Since diagnosis of RMS was not early considered in our patients, they were initially treated by a surgical resection [2]. Treatment is based on chemotherapy frequently followed by radiotherapy or surgical resection especially in cases where complete excision can induce mutilating surgery. Duration of chemotherapy is established on the basis of the previous European experience. It is of 22 weeks of VA chemotherapy for low-risk patients [10].

Multidisciplinary approach in the management of RMS with chemotherapy, surgery and/or radiotherapy has improved the prognosis with a five-year survival rate of 74–77% [8]. European groups tried to limit the use of radiotherapy to reduce side effects [10]. In our cases, retro auricular RMS was considered as a favorable site because the lesion was accessible to complete resection with an early diagnosis. An upfront completed resection was a viable option and leaded to a good long-term result.

The International Society of paediatric Oncology published data describing significant improvement in terms of remission rates in case of non-metastatic RMS in paediatric patients [5]. Side effects following treatment can be observed like chronic aural discharge, facial palsy or deformity with regional post-treatment radiation and patients requiring tarsorrhaphy or mandibular reconstruction. After treatment, all patients should be followed for tumor relapse and should monitor their treatment for side effects. The course of chemotherapy was not modified in our cases because of side effects. Both children are free of recurrence after 5 years of follow up.

## 4 Conclusion

Retro auricular RMS is rare. Early diagnosis is a challenge. Clinicians should be aware because of its ubiquitous distribution or diverse morphology. Biopsy should be performed rapidly in case of the slightest doubt.

Histopathological examination with immunohistochemical studies are required to achieve diagnosis. Imaging provides an assessment about the extension and has a key role in the initial staging and moreover during the follow up.

Treatment follows a rigorous international protocol associating surgery, chemotherapy and sometimes radiation therapy. Prognosis is good, as the site is accessible to a complete resection.

All patients require long term follow-up by a multidisciplinary team to exclude recurrence and to recognize and treat post-treatment complications.

## Declaration of Competing Interest

No conflict of interest.

No financial and personal relationship.

## Funding

No source of funding.

## Ethical approval

No ethical approval because no research studies.

## Consent

Written informed consent was obtained from both of the patient's parents for publication of this case report and accompanying images. A copy of the written consent is available for review by the Editor-in-Chief of this journal on request.

## Author contribution

M. ROUBAUD JC write the case report.

## Registration of research studies

It’s a case report.

## Guarantor

Pr MARIANOWSKI Rémi.

## Provenance and peer review

Not commissioned, externally peer-reviewed.

## References

[1] Chirat M., Dainese L., Fasola S., Couloigner V., Denoyelle F., Garabedian E.-N., Leboulanger N. (2016). Unusual outer ear swelling: childhood auricular rhabdomyosarcoma. Eur. Ann. Otorhinolaryngol. Head Neck Dis..

[2] Burrows N.P., Ratnavel R.C., Grant J.W., Cormack G.C., Pye R.J. (2014). Auricular embryonal rhabdomyosarcoma. Dermatology.

[3] Eksan M.S., Noorizan Y., Chew Y.K., Noorafidah M.D., Asma A. (2014). Rare embryonal rhabdomyosarcoma of external acoustic canal: a case report and literature review. Am. J. Otolaryngol..

[4] (2005). Protocol of the European Pediatric Soft Tissue Sarcoma Study Group. http://www.epssgassociation.it/index.php.

[5] Durve D.V., Kanegaonkar R.G., Albert D., Levitt G. (2004). Paediatric rhabdomyosarcoma of the ear and temporal bone. Clin. Otolaryngol. Allied Sci..

[6] Crozier E., Rihani J., Koral K., Cope-Yokoyama S., Rakheja D., O Ulualp S. (2012). Embryonal rhabdomyosarcoma of the auricle in a child. Pediatrics Int. Jpn. Pediatric Soc..

[7] Vankalakunti M., Das A., Rao N.K. (2006). Postauricular congenital alveolar rhabdomyosarcoma - a case report of an unusual entity. Diagn. Pathol..

[8] Singh G.B., Arora R., Kumar D., Jain M., Puri V. (2013). A rare case of congenital rhabdomyosarcoma with review of the literature. Case Rep. Otolaryngol..

[9] Skelton V.A., Goodwin A. (1999). Perinatal management of a neonate with airway obstruction caused by rhabdomyosarcoma of the tongue. Br. J. Anaesth..

[10] Bisogno G., Hawkins D.S. (2020). An unresolved issue in rhabdomyosarcoma treatment: the duration of chemotherapy. Pediatr. Blood Center.

[11] Ferlito A., Thomas Robbins K., Silver C.E., Hasegawa Y., Rinaldo A. (2009). Classification of neck dissections: an evolving system. Auris Nasus Larynx.

